# Revisiting High-Sensitivity Cardiac Troponin Abnormal Baseline Cutoffs: Implications for AMI Diagnosis in the Emergency Department

**DOI:** 10.3390/jcm14207308

**Published:** 2025-10-16

**Authors:** Kavithalakshmi Sataranatarajan, Madhusudhanan Narasimhan, Ishwar Daniel Chuckaree, Jyoti Balani, Ray Zhang, Rebecca Vigen, Alagarraju Muthukumar

**Affiliations:** 1 Department of Pathology, University of Texas Southwestern Medical Center, Dallas, TX 75235, USA; kavithalakshmi.sataranatarajan@utsouthwestern.edu (K.S.); madhusudhanan.narasimhan@utsouthwestern.edu (M.N.); jyoti.balani@utsouthwestern.edu (J.B.); ray.zhang@utsouthwestern.edu (R.Z.); 2 Division of Cardiology, Department of Internal Medicine, University of Texas Southwestern Medical Center, Dallas, TX 75235, USA; ishwar.daniel.chuckaree@emory.edu (I.D.C.); rebecca.vigen@utsouthwestern.edu (R.V.)

**Keywords:** Roche high-sensitivity cardiac Troponin T (hs-cTnT), Abbott high-sensitivity cardiac Troponin I (hs-cTnI), Chest pain, cardiac injury, acute Myocardial infarction (AMI)

## Abstract

**Background**: Current clinical guidelines recommend 52 ng/L as the abnormal baseline cutoff in high-sensitivity cardiac troponin (hs-cTn) algorithms for the rapid diagnosis of acute myocardial infarction (AMI). Though abnormal, this threshold is not AMI-specific, leading to extensive workups for many non-AMI chest pain patients, overutilization of resources, and emergency department (ED) overcrowding. Hence, the performance of this baseline abnormal cutoff was compared against the refined new thresholds for rapid AMI diagnosis in ED chest pain patients. **Methods**: We included ED chest pain patients with hs-cTnT and hs-cTnI levels simultaneously measured and clinical outcomes adjudicated by cardiologists. We performed receiver operating characteristics (ROC) analyses across various thresholds for diagnostic performance, including sensitivity, specificity, negative and positive likelihood ratios, and predictive values. Statistical analysis was carried out using Graphpad Prism 10, with *p* < 0.05 considered as significant. **Results**: In our study, 17 patients were adjudicated as AMI, and 682 patients were ruled out for AMI. In 15/17 AMI cases, baseline hs-cTn values far exceeded 52 ng/L. Notably, among non-AMI individuals, 140 (hs-cTnT) and 91 (hs-cTnI) also exceeded this cutoff. ROC analyses identified optimal abnormal cutoffs of 82 ng/L for hs-cTnT and 122 ng/L for hs-cTnI, which improved specificity without compromising sensitivity. Post-discharge follow-up at 1, 3, and 12 months for cardiovascular events supported these revised thresholds. **Conclusions**: Increasing the baseline abnormal value from 52 ng/L to 82 ng/L for hs-cTnT and to 122 ng/L for hs-cTnI in care pathways could reduce false positives with the potential to decrease unnecessary testing and alleviate long stays in the ED and resource management. Larger, diverse cohort studies are warranted to validate these findings.

## 1. Introduction

Current guidelines from the American Heart Association, American College of Cardiology, and European Society of Cardiology endorse high-sensitivity cardiac troponins (hs-cTns) as the preferred biomarker in emergency department (ED) patients presenting with suspected myocardial infarction [1,2]. Several streamlined diagnostic algorithms utilizing baseline hs-cTn values and delta changes from serial measurements have been proposed for the rapid diagnosis of AMI [1,2,3]. With the increasing adoption of hs-cTn testing in clinical practice by U.S. laboratories, there is an opportunity to evaluate the performance of various algorithms for AMI rule-in and rule-out [4].

Given ED overcrowding and the need for rapid triaging of non-AMI patients, the clinical focus is on promptly excluding non-AMI cases. This is mainly accomplished through the baseline rule-out cutoff values and delta changes in timed serial samples. The FDA defines 6 ng/L (hs-cTnT) and 5 ng/L (hs-cTnI) as the baseline cutoffs for rapid rule-out of AMI, besides requiring numerical reporting of cTn levels at or above the limit of quantitation (LoQ) [5,6].

A baseline abnormal cutoff is incorporated into Tn algorithms for the early diagnosis of AMI cases so that immediate medical attention and timely intervention can be provided to those critical patients. The current AMI care pathway algorithms incorporate an early abnormal baseline value of 52 ng/L as the initial decision point in their flow charts [7,8]. Earlier studies and international clinical guidelines endorsed using the 99th percentile of hs-cTn assays as a rapid abnormal cutoff for identifying true AMI cases in their algorithm [9,10]. This threshold corresponds to the 99th percentile value derived from a healthy reference population. However, variability exists between different assays and laboratories in the exact numeric value for the 99th percentile, leading to differences in specific cutoffs commonly used in clinical pathways. Several research groups have shown 52 ng/L to be a better early abnormal cutoff than the 99th percentile of hs-cTns [11,12,13]. Classification and regression tree (CART) analysis identified an optimal threshold value of 52 ng/L at presentation or absolute change in hs-cTnT within the first hour of 5 ng/L or higher to indicate a high probability of being a true AMI [14]. Based on these findings, current guidelines recommend 52 ng/L as the early abnormal cutoff value for both hs-cTnT and hs-cTnI tests. Our facility was one of the earliest facilities to implement the Roche fifth-generation TnT assay (hs-cTnT) in the U.S. Following the upgrade of our core laboratory automated chemistry system, we transitioned to the Abbott hs-cTnI assay. Throughout this changeover, the 52 ng/L cutoff continued to be used as an early abnormal cutoff for AMI rule-in across both assays.

Several studies have demonstrated that an elevated baseline abnormal hs-cTn value exceeding 52 ng/L is not uncommon in ED chest pain patients without MI, with such elevations often linked to sex, age, and kidney function differences [15,16,17,18]. Thus, although a 52 ng/L diagnostic threshold indicates abnormal troponin levels and may indicate high-risk, it is not specific enough to distinguish AMI exclusively from other causes of troponin elevation. Also, given the wide spectrum of patients encountered in the ED, with varying comorbidity burdens and age groups, an abnormal cutoff value derived from healthy individuals for hs-cTns may not adequately capture the normal variation seen in clinical practice. Therefore, it is critical to provide a clinically useful threshold where the assay ensures better alignment with patient heterogeneity and optimizes biomarker interpretation. Therefore, in the current study, we sought to evaluate whether the 52 ng/L cutoff remains the most effective diagnostic threshold at presentation for AMI diagnosis or whether refinements could enhance specificity without compromising sensitivity.

## 2. Materials and Methods

**Figure 1:**
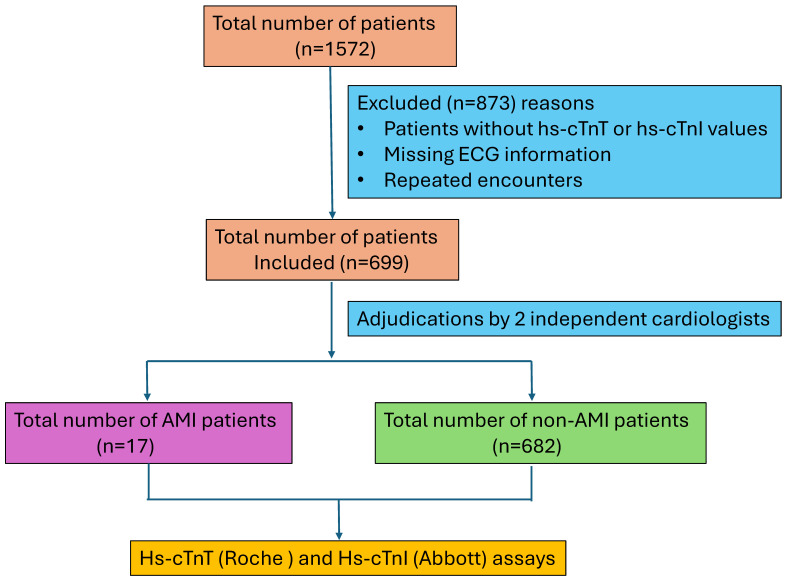
Flow diagram of the patients included in this study.

The institutional review board of the University of Texas Southwestern Medical Center (UTSW) approved this study, and it was performed in accordance with the Declaration of Helsinki. This retrospective cohort study was conducted in our care facility between May and September 2020 during our planned transition from Roche high-sensitivity cardiac troponin T (hs-cTnT) to Abbott high-sensitivity cardiac troponin I (hs-cTnI). Both markers are used in the detection of myocardial injury and the differential diagnosis of acute coronary syndromes. The hs-cTnT assay targets troponin subunit T and is manufactured by Roche Diagnostics, while the hs-cTnI assay targets troponin subunit I and is manufactured by other major vendors, such as Abbott and Siemens. Studies show comparable diagnostic performance for both assays, but hs-cTnT is elevated in renal dysfunction, while hs-cTnI remains less affected [19,20]. Adults > 18 years of age or older presenting to our university hospital ED and meeting the eligibility criteria were included in this study. The eligibility criteria included a. an initial encounter during the specified period, b. availability of the serial samples to complete the clinical decision pathway (CDP), c. ECG, and d. heart score diagnosis. Demographics, gender, and other clinical manifestations during the ED visit were obtained from the EPIC system. Patients missing hs-cTnT or hs-cTnI levels, ECG information, and repeated encounters were excluded from the analysis. Of the 1572 patients, only 699 patients were found to be eligible for the current study analysis, while the remaining samples were either missing a diagnosis or had repeated ED visits. A flow diagram of patients included in the study is shown in Figure 1. Simultaneous hs-TnT and hs-TnI testing was carried out on the initial presentation samples, with follow-up measurements at 1 h and 3 h, guided by clinical findings and clinician assessment [21]. Hs-cTnT measurements were performed on plasma samples collected in dipotassium EDTA, while the hs-cTnI assay used lithium heparin plasma [22,23].

### Clinical Diagnosis

All cases were adjudicated by 2 cardiologists based on the hs-cTn protocol and the fourth universal definition of myocardial infarction. Adjudication categories included Type I and Type II MI and acute or chronic myocardial injury [24,25]. HEART score, nonischemic ECG, and clinical symptoms were considered in the adjudication. Non-AMI cases mostly had secondary complications, such as chronic kidney disease, sepsis, hypertension, respiratory syndrome, and coronary heart disease. Receiver operating characteristic (ROC) curve analysis was conducted using hs-cTnT and hs-cTnI levels measured at presentation to identify the optimal abnormal cutoff for AMI. Diagnostic performance parameters, such as clinical sensitivity, specificity, positive and negative predictive values, as well as likelihood ratios, were calculated for various threshold cutoff values. Clinical outcomes, including mortality, myocardial infarction, and revascularization, were tracked longitudinally over 30 days, 90 days, and up to one year through EPIC electronic medical records. Statistical analyses were performed using GraphPad Prism 10, considering *p* < 0.05 as statistically significant.

## 3. Results

**Figure 2:**
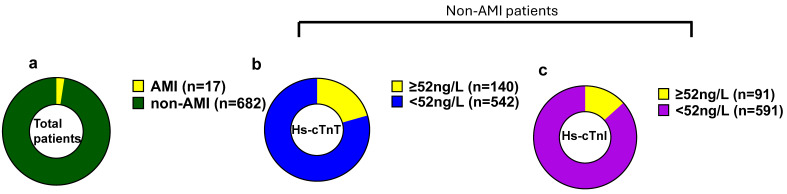
Hs-cTnT and hs-cTnI baseline values in ED patients with chest pain. Hs-cTn baseline values in ED patients with chest pain. (**a**). Total number of patients. (**b**). Baseline hs-cTnT levels in non-AMI patients. (**c**). Baseline hs-cTnI levels in non-AMI patients.

**Figure 3:**
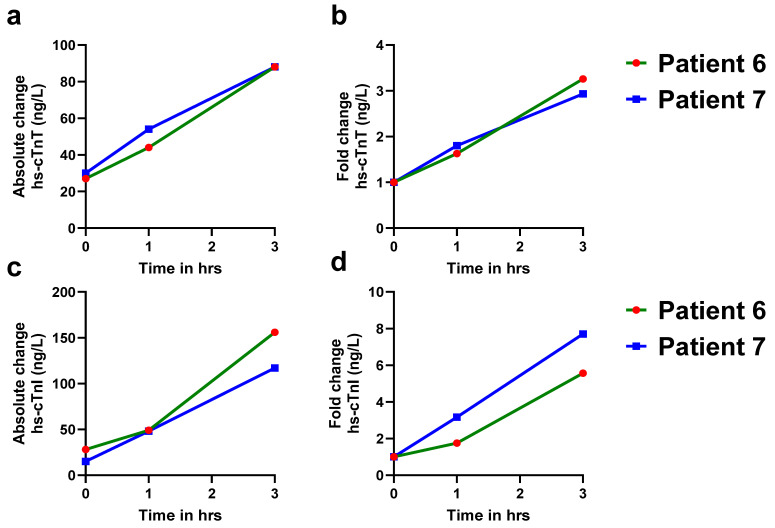
Serial measurements of hs-cTnT and hs-cTnI levels in AMI patients with baseline values < 52 ng/L. A more than 20% increase in hs-cTn levels at 1 h and 3 h in both patients indicates dynamic troponin kinetics, which are more consistent with acute myocardial infarction (AMI). Baseline hs-cTnT and hs-cTnI levels in AMI patients. (**a**). Absolute change in hs-cTnT levels in patient #6 (green solid line) and patient #7 (blue solid line) at 0, 1, and 3 h. (**b**). Fold change in hs-cTnT levels in patient #6 (green solid line) and patient #7 (blue solid line) at 0, 1, and 3 h. (**c**). Absolute change in hs-cTnI levels in patient #6 (green solid line) and patient #7 (blue solid line) at 0, 1, and 3 h. (**d**). Fold change in hs-cTnI levels in patient #6 (green solid line) and patient #7 (blue solid line) at 0, 1, and 3 h.

At our university hospital ED, hs-cTn values of ≥52 ng/L are designated as abnormal for AMI in the care pathways. Among 699 patients presenting with chest pain, the majority (97.4%) were diagnosed as non-AMI cases, while 2.4% were found to be true positives for AMI (Figure 2a). Over half of the ED patients with acute chest pain had hs-cTn levels below 52 ng/L for both assays (Figure 2b,c). Although 26% of patients for hs-cTnT and 15% of patients for hs-cTnI exceeded the abnormal cutoff threshold of 52 ng/L, the majority of these patients were non-AMI patients. After adjudication, only 17 patients were confirmed for AMI. Of these patients, 15 had hs-cTn levels exceeding 52 ng/L at presentation, while 2 patients (patient numbers 6,7) initially tested below this threshold (Table 1). However, both these patients exhibited significant increases in troponin levels over time, with delta changes at 3 h post-presentation indicative of AMI or myocardial injury (Figure 3a,c).

**Figure 4:**
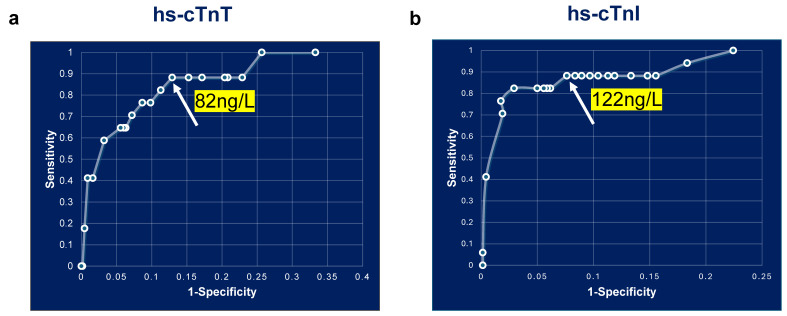
ROC curve analysis of baseline hs-cTnT and hs-cTnI measurements. ROC curve analysis of baseline hs-cTn measurements. (**a**). hs-cTnT and (**b**). hs-cTnI.

A comparison of ROC curves for hs-cTnT and hs-cTnI demonstrated that both assays had a baseline sensitivity of 88% at the cutoff of 52 ng/L. For hs-cTnT, this sensitivity remained constant up to 82 ng/L (Figure 4a), and for hs-cTnI, it remained constant up to 122 ng/L (Figure 4b). Applying an 82 ng/L cutoff for hs-cTnT improved specificity from 79% (95% CI; 0.77, 0.82) to 87% (95% CI; 0.84,0.89). Similarly, raising the hs-cTnI cutoff to 122 ng/L increased specificity to 92% (95% CI: 0.90, 0.94). Notably, these increases in the cutoff improved specificity while maintaining similar sensitivity as that of 52 ng/L (Table 2). The negative predictive value (100%) and negative likelihood ratios were unchanged across all thresholds. Although sensitivities remained unchanged at the higher cutoffs, positive predictive value abnormal for AMI rose to approximately 1.6-fold for both hs-cTnT (0.09 (52 ng/L) vs. 0.15 (82 ng/L)) and hs-cTnI (0.14 (52 ng/L) vs. 0.22 (122 ng/L)) (Table 2). Importantly, we observed no corresponding change in positive likelihood ratios, suggesting the higher thresholds result in fewer false-positive cases at presentation in the ED. We also noticed that hs-cTnI has better sensitivity and specificity compared to hs-cTnT, consistent with reports from previous studies [9,25,26].

**Figure 5:**
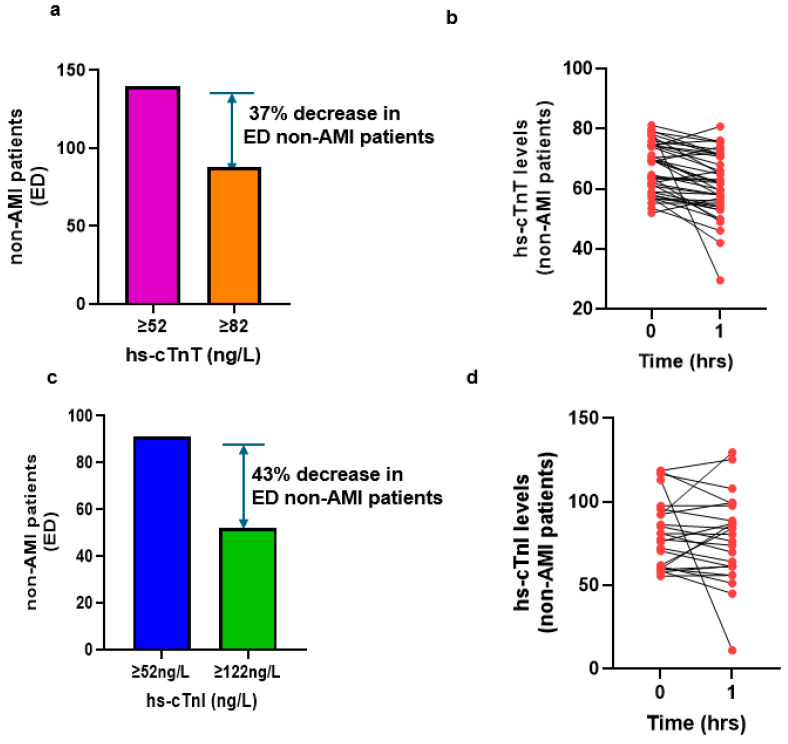
Number of non-AMI patients in the newly derived baseline abnormal cutoff and absolute changes in 1 h. Non-AMI patients in the ED with chest pain. (**a**). Baseline hs-cTnT levels ≥ 52 ng/L versus baseline ≥ 82 ng/L; (**b**). 1 h absolute change in hs-cTnT levels; (**c**). baseline hs-cTnI levels ≥ 52 ng/L versus baseline ≥ 122 ng/L; (**d**). 1 h absolute change in hs-cTnI levels.

Next, we assessed whether applying the refined abnormal cutoffs of 82 ng/L for hs-cTnT and 122 ng/L for hs-cTnT could reduce the proportion of non-AMI chest pain patients flagged as abnormal for AMI in the ED. Compared to the existing threshold of 52 ng/L for AMI, these elevated cutoffs reduced false-positive cases by 37% for hs-cTnT and 43% for hs-cTnI (Figure 5a,c). We checked 0/1 h absolute changes in patients with baseline hs-cTn values between 52 and 82 ng/L (hs-cTnT) and 52 and 122 ng/L (hs-cTnI). (Figure 5b,d). We analyzed the delta change in patients who had 1 h hs-cTn values measured. No patients exhibited a delta change of more than a 20% increase in hs-cTnT levels (Figure 5b), while three patients exceeded a delta change of more than 20% in hs-cTnI levels (Figure 5d). Nevertheless, these cases were subsequently confirmed by cardiologists as nonischemic myocardial injury patients.

To validate the effectiveness and assess the clinical utility of the revised abnormal cutoffs in non-AMI patients, we conducted a follow-up review of ED return visits in chest pain patients with baseline troponin levels between 52 and 82 ng/L for hs-cTnT and 52 and 122 ng/L for hs-cTnI. We documented reasons for the ED visits at 1-, 3-, and 12-month post-discharge (Table 3). There were 52 patients with baseline hs-cTnT values between 52 and 82 ng/L and 39 patients with baseline hs-cTnI values between 52 and 122 ng/L. No cardiac deaths were reported among those patients followed up after 1 month and 12 months (Table 3). However, one case was detected due to comorbidities of renal failure, pneumonia, and atrial fibrillation after 3 months of follow-up. The majority of the patients who re-visited the ED were due to non-cardiac-related issues, such as COPD, pneumonia, sepsis, hypertension, renal failure, end-stage renal disease, stroke, and cholecystitis. A very low incidence of cardiac-related events was observed after 1 month (0.2% for hs-cTnT; 0.5% for hs-cTnI), 3 months (0.1% for hs-cTnT; 0.3% for hs-cTnI), and 12 months (0.3% for both hs-cTnT and hs-cTnI).

## 4. Discussion

In the current study, the optimal diagnostic abnormal thresholds for AMI were identified as 82 ng/L for the hs-cTnT assay and 122 ng/L for the hs-cTnI assay. Applying these revised diagnostic abnormal values at presentation in the ED decreased the detection of false-positive cases. Integrating these revised abnormal thresholds along with a 1 h delta troponin change criterion provided a good strategy for confirming true AMI cases in the ED.

Over the past few decades, high-sensitivity cardiac troponins have revolutionized the diagnosis of AMI. Numerous research groups have developed and validated diagnostic algorithms, defining rule-out/rule-in for AMI in ED patients with chest pain [9,12,14,27,28]. The 52 ng/L rule-in/abnormal cutoff is part of a broader strategy that typically integrates serial measurements and evaluates dynamic changes in troponin levels over time. This was adopted from the European Society of Cardiology (ESC) rule-in criteria for acute coronary syndrome (ACS), which balanced the sensitivity and specificity—high enough to rule-in AMI in many cases, yet low enough to identify early cardiac injury. Within the ESC 0/1 h algorithm, 52 ng/L is applied to both hs-cTnT and hs-cTnI assays. For hs-cTnT, the abnormal criterion is either an initial value ≥ 52 ng/L or a 1 h rise of ≥ 5 ng/L [10]. Although this cutoff has been validated in multiple studies and incorporated into ESC guidelines, the hs-cTnT 0/1 h protocol allows for accurate early triage in only about 75% of patients, with 60% ruled out for MI and 15% ruled in [29]. In another study, the same cutoff of ≥52 ng/L (hs-cTnT) identified 26% of chest pain patients in a high-risk category, of which only 7% had an MI [30]. Adding to this complexity, in U.S. EDs, the troponin testing practices are inconsistent; in one large analysis of 97,085 ED visits from the National Hospital Ambulatory Medical Care Survey (NHAMCS), only 7% of patients had biomarker testing, and two-thirds of these patients lacked chest pain at presentation, with ACS prevalence below 2% [31].

All AMI diagnostic algorithms share a central goal to ensure no true infarction case is overlooked. In Europe, these algorithms are well standardized with respect to diagnostic cutoff values and triage protocols. In contrast, many U.S. facilities remain in transition from contemporary troponin assays to hs-cTns as the preferred biomarker for ED chest pain patients. A multicenter pre–post cohort study of 17,384 patients revealed that using hs-cTns within the HEART pathway improved AMI detection and reduced healthcare utilization compared with conventional assays [32]. In the U.S. clinical setting, FDA-approved hs-cTn assays must report results only when values are at or above the limit of quantification (LoQ). For AMI rule-in, guidelines require the detection of a rise and fall pattern with at least one value above the 99th percentile URL [25,33]. The ESC recommends an absolute value of 52 ng/L as an abnormal cutoff for AMI indication [34], consistent with CART analysis finding either 52 ng/L or a ≥ 5 ng/L change in 1 h [14]. This guideline recommends that the abnormal value of 52 ng/L be thoroughly evaluated in different U.S. populations [35,36]. Our ROC analysis identified 82 ng/L (hs-cTnT) and 122 ng/L (hs-cTnI) as optimal abnormal thresholds at presentation for diagnosing AMI. At this cutoff, the sensitivity remained comparable to that of 52 ng/L, while the specificity was improved. Importantly, these refined thresholds shifted patient triage patterns compared to 52 ng/L and have the potential to optimize ED resource use, cost, and workflow in chest pain management.

ACC recommendations emphasize measuring hs-cTns serially to determine the change in concentration at 1 and 3 h after initial presentation. An increase of more than 20% is suggested to support a diagnosis of AMI, while changes below this threshold often indicate resolving AMI [37,38]. In our study, AMI patients consistently demonstrated more than 20% rises in hs-cTn levels, whereas most non-AMI patients with baseline values below the newly proposed thresholds (52–82 ng/L for hs-cTnT, 52–122 ng/L for hs-cTnI) showed <20% change or even a decrease after 1 h. Post-discharge follow-up data at 1, 3, and 12 months in patients within this intermediate range revealed minimal or negligible subsequent cardiac events. This suggests that a single hs-cTn test at presentation may be adequate for many non-AMI cases, though adding a 1 h delta measurement offers reassurance in excluding non-AMI cases and improving diagnostic confidence. However, the HEART score combined with the 0/1 h troponin algorithm is a strategy practiced in many EDs for the diagnosis of adverse cardiac events [39].

Beyond AMI, elevated hs-cTn levels can occur in a variety of clinical settings, including, but not limited to, sepsis, stroke, CKD, left ventricular hypertrophy, hypertension, advanced age, and acute respiratory distress syndrome [40,41,42,43,44,45,46]. Consistent with this, our findings indicate that 52 ng/L is not specific enough to rule out non-AMI, as many ED patients with chest pain have comorbidities, such as CKD, CHD, myopathies, and sepsis. Studies have shown a 2.5–2.8-fold change in delta values to perform relatively better in ruling in patients with comorbidities such as CKD [47]. Emerging evidence suggests that optimal abnormal cutoff values may vary depending on underlying disease states; for example, a threshold of 127 ng/L has been reported to offer good specificity for detecting obstructive coronary artery disease [48]. Elevated hs-cTn levels exceeding 100 ng/L are frequently observed in septic shock patients [49]. In the chronic renal insufficiency cohort study (CRIC), the 99th percentile for hs-cTnT was 126 ng/L [50]. Integrating troponin levels along with serial measurement, underlying disease conditions can differentiate acute from chronic elevations regardless of baseline troponin levels. The tailoring of diagnostic abnormal cutoffs should include patient-specific factors along with overall clinical status to enhance diagnostic accuracy.

## 5. Strengths and Limitations

Few reports exist comparing Roche hs-cTnT and Abbott hs-cTnI results from the same sample timed. Similarly, few studies have addressed the abnormal cutoff values for hs-cTn, as many U.S. facilities are still transitioning from conventional cTn assays to hs-cTn. In this report, we review the existing evidence for both hs-cTn assays, focusing on hs-cTn levels obtained at presentation combined with a 20% increase in 1 h delta change as abnormal criteria for AMI. Our analysis targets the baseline abnormal threshold specifically, given its critical importance in facilitating immediate intervention for true AMI cases. This approach supports rapid prioritization of true AMI patients while enabling timely triage of non-AMI individuals, helping to mitigate ED length of stay and optimizing resource management.

This study has important limitations to consider. Foremost, this is a monocentric study, which may limit the generalizability of our proposed AMI rule-in/abnormal diagnostic value to broader and more heterogeneous patient populations. Multicenter research across different regions and healthcare systems will be critical for confirming and standardizing these diagnostic criteria. Second, the study period coincided with the COVID pandemic, during which a considerable number of patients presented with comorbidities, such as shortness of breath, kidney disease, and sepsis, unrelated to AMI. Finally, the modest sample size reduces the statistical power, highlighting the need for larger studies to confirm diagnostic performance and support clinical adoption.

## 6. Conclusions

Given that hs-cTn levels are influenced by multiple conditions, our study identifies 82 ng/L for hs-cTnT and 122 ng/L for hs-cTnI as optimized baseline abnormal cutoffs for AMI diagnosis in the U.S. population. Large-scale studies within other U.S. facilities are essential to confirm and standardize these thresholds that can streamline ED triage and expedite definitive care for AMI patients.

## Figures and Tables

**Table 1:** hs-cTnT and hs-cTnI levels at 0,1, and 3 h in AMI patients.

Patient	hs-cTnT (ng/L)			hs-cTnI (ng/L)			ADJUDICATION
	**0 h**	**1 h**	**3 h**	**0 h**	**1 h**	**3 h**	
1	7499	8471	NA	5000	5000	NA	NSTEMI Type 1
2	95.91	129.9	NA	1052	1151	NA	NSTEMI Type 1
3	215	259	290	1283	2011	2488	NSTEMI Type 1
4	210	352	NA	3490	4169	NA	NSTEMI, type I but MINOCA and COVID pos
5	1181	NA	NA	4392	NA	NA	NSTEMI unclear if Type I vs. Type II
6	27	44	88	28	49	156	NSTEMI, Type I but MINOCA
7	30	54	88	15	48	117	NSTEMI Type I, MINOCA
8	911.3	NA	NA	1458	NA	NA	NSTEMI, unclear type I vs. II (favor Type II)
9	91.09	262.8	NA	130	1034	NA	Type 2: NSTEMI
10	868.3	876.2	NA	7739	7093	NA	NSTEMI Type 1
11	112.7	106.6	NA	355	499	NA	NTSTEMI Type I
12	558	903.7	NA	1879	9086	NA	STEMI Type 1
13	158.4	164.1	NA	233	179	NA	Type 2: NSTEMI due to demand ischemia
14	222.1	NA	NA	1018	NA	NA	Type I NSTEMI
15	614.2	643.2	NA	4111	2824	NA	Type I NSTEMI
16	7251	NA	NA	5000	NA	NA	Type I NSTEMI
17	123	406	NA	596	4900	NA	NSTEMI Type 1

**Table 2:** Diagnostic performance of the baseline abnormal cutoff and newly derived abnormal cutoff of the hs-cTnT and hs-cTnI assays.

Assay	Cutoff	Sensitivity	Specificity	PPV	NPV	LR+	LR−
Hs-cTnT	>52	0.88 (0.73, 1.03)	0.79 (0.77, 0.82)	0.09 (0.05, 0.14)	0.99 (0.99, 1.0)	4.32 (3.45, 5.44)	0.14 (0.04, 0.54)
	>82	0.88 (0.73, 1.03)	0.87 (0.85, 0.90)	0.15 (0.08, 0.21)	0.99 (0.99, 1.0)	6.84 (5.27, 8.88)	0.14 (0.04, 0.50)
Hs-cTnI	>52	0.88 (0.73, 1.04)	0.87 (0.84, 0.89)	0.14 (0.08, 0.20)	1.0 (1.0, 1.0)	6.61 (5.10, 8.56)	0.14 (0.04, 0.5)
	>122	0.89 (0.73, 1.03)	0.92 (0.90, 0.94)	0.22 (0.12, 0.32)	1.0 (1.0, 1.0)	11.6 (8.5, 15.83)	0.13 (0.03, 0.47)

**Table 3:** Post-discharge clinical follow-up of non-AMI patients with baseline Tn values between 52 ng/L and newly derived cutoff ranges.

		1st ED Visit		1 Month		3 Months		12 Months	
		TnT	TnI	TnT	TnI	TnT	TnI	TnT	TnI
	**no of patients**	52	39	15	13	14	12	23	13
**Reason**	**cardiac related**	14	13	3	6	2	4	6	4
	**non-cardiac**	38	26	12	7	12	8	17	9
**Mortality**	**cardiac related**	0	0	0	0	1	0	0	0
	**non-cardiac**	0	0	3	4	2	2	7	8

## Data Availability

The original contributions presented in this study are included in this article. Further inquiries can be directed to the corresponding author.
